# Supplementary material for: Genetic Diversity and Structure of Persian Walnut (Juglans regia L.) in Pakistan: Implications for Conservation
Source: Plants (Basel). 2022 Jun 22;11(13):1652. doi: 10.3390/plants11131652 (PMC9269025; doi:10.3390/plants11131652)
Supplement: Supplementary file 1 [file plants-11-01652-s001.zip › plants-1677739-supplementary figures.pdf]

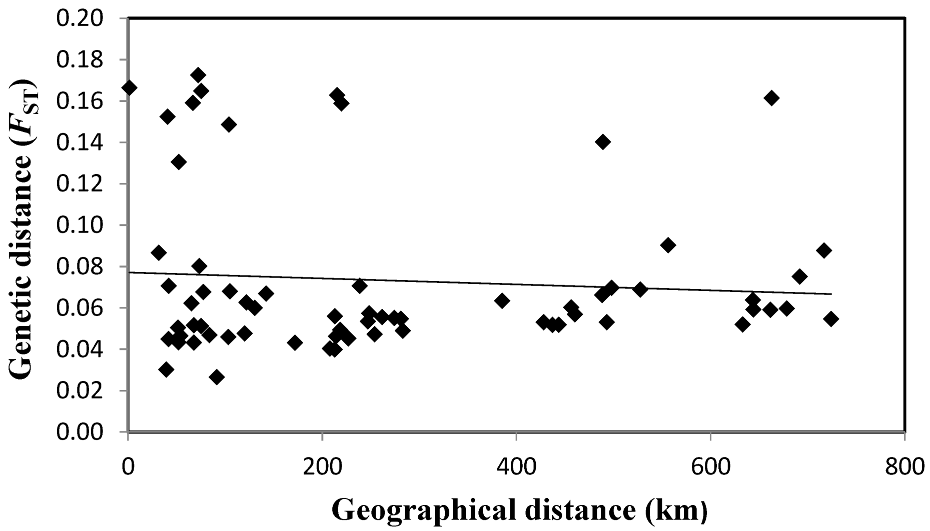

Figure S1. Correlation of geographic distance (in kilometers) and genetic distance (pairwise F_{ST}) among 12 populations of *J. regia*, including regression line (r = 0.14, p = 0.22 at 1000 randomization).

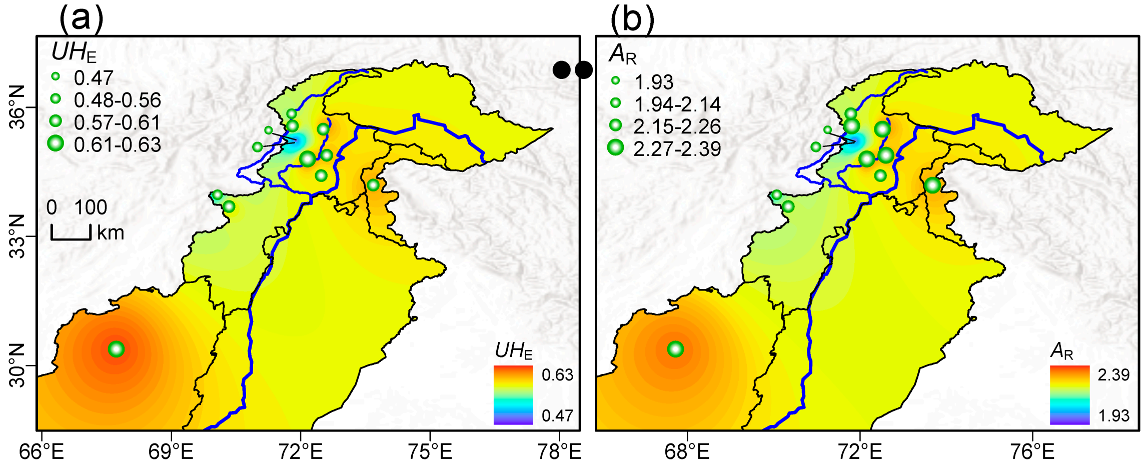

Figure S2. Spatial genetic diversity of 12 populations of Pakistan walnut: (a) Unbiased expected heterozygosity (UH_E), (b) allelic richness (A_R).

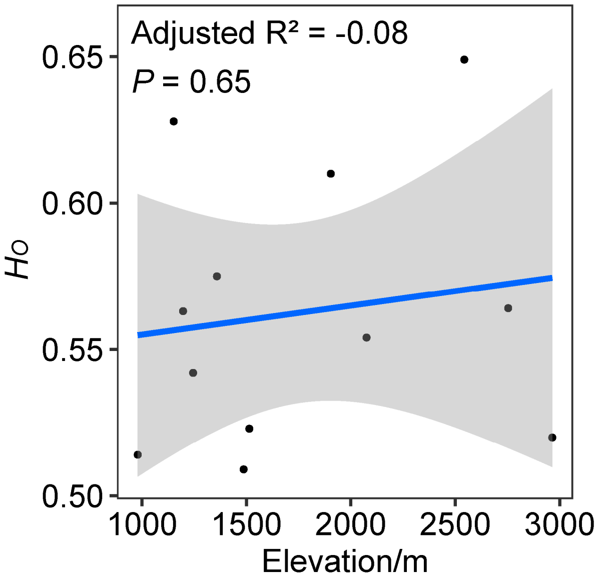

Figure S3. Relationship between observed heterozygosity and elevation for 12 populations of walnut in Pakistan.
